# Sensory-cognitive associations are only weakly mediated or moderated by social factors in the Canadian Longitudinal Study on Aging

**DOI:** 10.1038/s41598-019-55696-5

**Published:** 2019-12-23

**Authors:** Anni Hämäläinen, Natalie Phillips, Walter Wittich, M. Kathleen Pichora-Fuller, Paul Mick

**Affiliations:** 1 School of Optometry, Université de Montréal, Montreal, Canada; 2 Department of Psychology, Concordia University, Montreal, Canada; 3 Department of Psychology, University of Toronto, Toronto, Canada; 4 Department of Surgery, University of Saskatchewan, Saskatoon, Canada

**Keywords:** Human behaviour, Epidemiology, Risk factors

## Abstract

Sensory and cognitive function both tend to decline with increasing age. Sensory impairments are risk factors for age-related cognitive decline and dementia. One hypothesis about sensory-cognitive associations is that sensory loss results in social isolation which, in turn, is a risk factor for cognitive decline. We tested whether social factors are associated with cognitive and sensory function, and whether sensory-cognitive associations are mediated or moderated by social factors. We used cross-sectional data from 30,029 participants in the Canadian Longitudinal Study of Aging, aged 45–85 years, who had no reported cognitive impairment or diagnosis of dementia. We found strong independent associations of self-reported social variables with hearing (pure-tone audiometry), vision (pinhole-corrected visual acuity), and executive function and weaker associations with memory. The moderating and mediating effects of social variables on sensory-cognitive associations were weak and mostly non-significant, but social factors could be slightly more important for females and older people. Partial retirement (relative to full retirement or not being retired) may have protective effects on cognition in the presence of hearing loss. These findings confirm the association between social factors and sensory and cognitive measures. However, support is weak for the hypothesis that social factors shape sensory-cognitive associations.

## Introduction

Cognitive and sensory impairments are frequently comorbid in older adults^1–9^. Hearing loss has been identified as the most potentially modifiable mid-life risk factor for dementia^10^. Although recent meta-analyses found no overall association between visual acuity and higher-level cognitive performance^11,12^, certain ocular pathologies have been associated with Alzheimer’s disease^6,13^. Several hypotheses have been formulated concerning the potential bases of these sensory-cognitive associations^4,5,14,15^. Cognitive decline has been hypothesized to result from long-term sensory deprivation, possibly because cognitive health is compromised by reduced social activity due to sensory impairments. This is consistent with evidence suggesting that social isolation increases the risk of cognitive decline and dementia^10,16^. However, such associations are nuanced; for example, the association between sensory and cognitive function may depend on cognitive domain^17^ and the effects of social isolation may be sex-specific^18,19^. Furthermore, sensory-social-cognitive associations have primarily been examined in people with cognitive impairments (but see^9^). Importantly, knowledge of how social factors may mediate links between sensory and cognitive aging in cognitively healthy populations can inform strategies for dementia prevention and/or early detection.

To examine sensory-cognitive-social associations, we used baseline cognitive, sensory, social, and health data from community-dwelling older adults who participated in the Canadian Longitudinal Study of Aging (CLSA,^20^). They were deemed to be cognitively normal and had no self-reported diagnosis of dementia. Previously, we demonstrated a positive relationship between the average audiometric hearing threshold and both memory and executive function, as well as an association of better visual acuity with better executive function (Phillips *et al*. unpublished). We have also found associations between self-report measures of sensory function and social factors (participation in social activities; size of social network; loneliness; social support) in the CLSA Comprehensive Cohort^21^ and in the CLSA Tracking Cohort^22^. Here, we test whether

1. cognitive measures are independently associated with measures of social factors;
2. previously observed sensory-cognitive associations (Phillips *et al*. unpublished) remain significant after controlling for social factors; and
3. sensory-cognitive associations are moderated or mediated by social factors.

## Methods

### Study population

We used the first wave of data (collected 2012–2015, released in 2017) from the CLSA Comprehensive Cohort. These cross-sectional data included behavioral measures of sensory and cognitive performance, and self-report measures of social participation and health. Details on the study design, choice of measurements and descriptions of participant characteristics are provided elsewhere^22–25^. Participants aged 45–85 years were recruited using provincial health registries and random-digit phone dialling. Exclusion criteria included living in institutions, living on First Nations reserves, full-time service in the Canadian Armed Forces, inability to respond in English or French, and cognitive impairment suspected by CLSA staff. Here, we also excluded anyone with a self-reported diagnosis of dementia (N = 68), resulting in a sample size N = 30,029 participants. Participants visited one of eleven data collection sites where sensory and cognitive measures were obtained and information on health and lifestyle was gathered by interview. Informed consent was obtained from all participants. Descriptive data for all variables used in this paper are presented in Table S1 (Appendix).

The present project received ethics approval by the University of British Columbia (#H15-00430) and the University of Montreal (#17-063-CERES-D). The research was performed in accordance with the relevant guidelines and regulations.

### Measures of sensory and cognitive function

We used previously described behavioral measures of hearing and vision^21,25^. Vision was measured as the better-seeing eye pinhole-corrected visual acuity (VA; reported in logMAR). Hearing was measured as the better-hearing ear pure-tone average (BPTA; reported in dB HL) across four frequencies: 1000, 2000, 3000 and 4000 kHz.

To quantify cognitive function, we used previously developed cognitive scores (Phillips *et al*. unpublished). Executive function (PC1) and memory (PC2) scores were derived from a principal component analysis of the test scores on five cognitive tests: Mental Alternation Test, Animal Fluency test, Controlled Oral Word Association Test, Stroop test, and Rey Auditory Verbal Learning Test with immediate and 5-minute recall; the choice and administration of these tests are described elsewhere^24^. Higher PC scores indicate better executive function and memory. It should be noted that some cognitive tests were administered in a way that required sufficient vision (Stroop test) or hearing (Rey Auditory Verbal Learning Test) to perform, and these tests were not completed by some participants with a significant uncorrected impairment in the corresponding sense. However, more minor difficulties seeing or hearing could have some confounding effect on the cognitive measures. For a thorough treatment of this possible issue, see (Phillips *et al*. unpublished).

### Social factors

The CLSA collected self-report data on several social factors, including the scope, opportunities for, and perceived quality of social engagement. The scope of social engagement was measured by the number of types of social activities the individual participated in with others (count of social activities: volunteer work; family/friend events (outside of the household); church/religious; sports/physical; educational/cultural; service club; community/professional association; other recreational activity), frequency of participation in social activities (“Never in past year”; “At least once in past year”; “At least monthly”; “At least weekly”; “Daily”), and social network index (SNI; count of the types of social interactions at least every 1–2 weeks over the past year with: children, other close family members, friends, neighbours, work colleagues, schoolmates, fellow volunteers, members of non-religious community groups, members of religious groups; being married or in a domestic partnership). Indicators of independence and everyday environment associated with social opportunity included driving status (driving at least occasionally vs. not driving); Life Space Mobility Composite index^26^ (LSI; compound score of frequency, independence, and distance moved in/from home within the past four weeks); retirement status (not retired, partly, or completely retired); and living situation (living alone vs. with others). Perception of the quality of social engagement was measured with self-reported measures of social support availability (composite score of the perceived availability of emotional/informational, tangible, affectionate social support; positive social interaction), loneliness (feeling lonely at least once in the past week), and desire for more social participation (wanting more social/recreational/group activities in the past year).

### Health and socioeconomic covariates

Socioeconomic factors were included as covariates: age (45–85 years), sex (50.9% female), cultural background (“white” vs. “other”), and annual household income bracket. We included modifiable risk factors for dementia identified in a recent synthesis paper^10^: level of education, body mass index, and self-reports of hypertension and diabetes diagnoses, smoking habits, head injuries (self-reported number of head injuries dichotomized to “at least one head injury” vs. “no head injuries”), and nutritional risk (derived from responses to dietary habits). We controlled for cognitive test language (English; French) because cognitive test results in the CLSA differed depending on test language (^24^; Phillips *et al*. unpublished).

### Data analyses

Missing data for all variables were imputed with multivariate, multiple imputation methods as detailed previously (Phillips *et al*. unpublished; Appendix). Model estimates were adjusted for the uncertainty arising from missing data. We applied inverse probability weights provided by CLSA (https://www.clsa-elcv.ca/doc/1041) to adjust for population representation of the participants based on sex, age, and province of residence.

To test whether cognitive measures were independently associated with social factors, we calculated correlations between all social, sensory, and cognitive measures. To confirm these associations and assess whether previously observed sensory-cognitive associations (Phillips *et al*. unpublished) remain significant after controlling for social factors, we built two multiple regression models of the direct effects of social variables on cognition. All risk factors and covariates were included simultaneously in separate models for executive function and memory.

We used the results from these two main-effects models (with either executive function or memory as the response variable) as a basis for testing the moderating (whereby social variables alter the strength of the sensory-cognitive associations) and mediating (whereby the effect of sensory function on cognition is hypothesized to be indirectly caused by their associations with social variables) effects of the social variables on the sensory-cognitive associations^27^. To examine whether social factors moderate any sensory-cognitive associations, one at a time, we added an interaction term for each social factor with either hearing or vision to each main-effects model. Thus, a total of 40 separate models were created to test the moderating effect of each of the ten social factors on each sensory-cognitive association (vision-executive function; hearing- executive function; vision-memory; hearing-memory). We conducted exploratory analyses of the potential mediation effects of social variables by comparing the effect sizes of vision and hearing on executive function and memory in the main-effects model, with an otherwise identical model that excluded all social variables (i.e., including only vision, hearing, and all control variables). To assess the possibility that the importance of social mediation differs in males and females or younger and older age groups, we also examined the associations separately for the sexes and for the younger (ages 45–64) and older subgroups (ages 65–85) of the population. We report the absolute and % change in effect size (i.e., the reduction in direct effect size of vision and hearing on cognition when accounting for social factors) and approximate R^2^ to estimate the total indirect effect of social factors on the sensory-cognitive associations. The statistical significance of the difference in effect sizes between the nested models (with and without social variables) was estimated with Z-tests. All effect sizes (β) reported in the tables are derived from the full regression models (i.e., they were corrected for all covariates).

All analyses were conducted in Stata version 15.1.

## Results

### Independent associations of sensory, cognitive, and social variables

**Figure 1:**
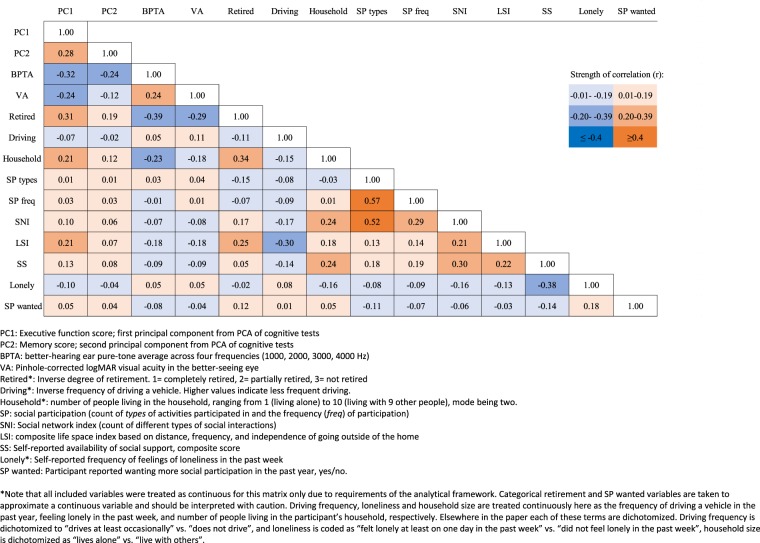
Correlation matrix of sensory, cognitive, and social variables used in the study. The legend indicates a color code for the strength of the correlation coefficient. The correlations (Pearson’s r) were calculated by transforming a covariance matrix of multiple imputation data using an expectation maximization algorithm to include all available data in the estimation, requiring all variables to be treated as continuous in this table only (see Appendix for details).

We observed correlations of r < |0.4| for each of the social variables with vision, hearing, and both cognitive domains (executive function and memory), and among the different social variables (males and females grouped together; Fig. 1). There were moderate (r = 0.4–0.6), positive correlations for the number of different types of social activities with participation frequency and SNI.

Multiple regression analyses (Table 1) confirmed generally small but statistically significant associations between executive function (PC1) and all social variables, apart from social participation frequency. Specifically, better executive function was associated with driving at least occasionally, living alone, being partially retired, participating in more different types of activities, a smaller SNI, a larger LSI, more perceived social support, not feeling lonely in the past week, and a desire for more social participation in the past year. The non-significant effect of participation frequency (and possibly the negative effect of SNI) on executive function may be partially due to the moderate correlation between social participation types and frequency (Fig. 1) such that the variance associated with social participation may be captured by the number of types of participation rather than by participation frequency. A higher memory (PC2) score was associated with being partially retired, driving, a *lower* LSI, and more social support.

**Table 1:** Predictors of cognitive function (PC1 (Executive function) and PC2 (Memory)) using the main effects model that includes all social variables.

		PC1				PC2			
		B	SE	t	P	B	SE	t	P
Hearing threshold	BPTA (10 dB HL)	−0.077	0.009	−8.960	**<0.001**	−0.046	0.008	−5.980	**<0.001**
Visual acuity	Pinhole-corrected logMAR	−0.785	0.074	−10.670	**<0.001**	−0.029	0.067	−0.440	0.660
Age	Years	−0.039	0.002	−25.280	**<0.001**	−0.029	0.001	−20.300	**<0.001**
Retirement status	fully retired	Ref.				Ref.			
	partly retired	0.158	0.030	5.260	**<0.001**	0.072	0.030	2.420	**0.016**
	not retired	0.050	0.028	1.780	0.075	−0.036	0.026	−1.350	0.177
Driving status	Drives at least occasionally	0.163	0.043	3.780	**<0.001**	0.092	0.039	2.360	**0.019**
Living arrangement	Lives alone	0.130	0.027	4.820	**<0.001**	0.020	0.026	0.790	0.431
Social participation	Participation types	0.037	0.008	4.480	**<0.001**	0.008	0.008	0.910	0.365
	Participation frequency	−0.023	0.018	−1.290	0.196	−0.009	0.018	−0.520	0.603
	Network index	−0.009	0.007	−1.240	0.215	0.005	0.008	0.650	0.517
Life space	Life space index	0.002	0.001	4.010	**<0.001**	−0.002	0.001	−2.970	**0.003**
Social support	Perceived support	0.002	0.001	3.190	**0.001**	0.002	0.000	3.490	**<0.001**
Loneliness	Sometimes-all the time (>1d)	−0.071	0.024	−2.980	**0.003**	−0.042	0.023	−1.830	0.068
Wanted more social participation	Yes	0.045	0.018	2.530	**0.011**	0.008	0.018	0.460	0.644
Intercept		3.596	0.147	24.400	**<0.001**	6.396	0.143	44.760	**<0.001**

Separate models were run for PC1 (Executive function) and PC2 (Memory). Significant results at P < 0.05 are shown in bold. Both models controlled for age, education, income, sex, cultural background, test language, hypertension, nutritional risk, diabetes, head injuries, smoking status and body mass index (full model outputs in Appendix, Table S2).

### Sensory-cognitive associations remain after correcting for social factors

The models controlling for social factors and health risk factors confirmed the previously described sensory-cognitive associations (Phillips *et al*. unpublished). Specifically, greater executive function scores were associated with better hearing and vision, and greater memory scores were associated with better hearing. The estimates for the effects of sensory and social variables on executive function and on memory are shown in Table 1; for full models see Appendix (Tables S1 and S2).

### Moderation and mediation of sensory-cognitive associations by social factors

Despite the direct associations between social and cognitive variables, and between sensory and cognitive function, we found very limited evidence of social variables moderating the associations between cognitive and sensory function (Table 2). The only statistically significant, but weak interaction effects on executive function were those of hearing with retirement and with SNI. Specifically, partial retirement was associated with better executive function for a given hearing threshold, relative to those in full retirement. A higher social network index (SNI) was associated with a stronger *negative* association between executive function and hearing threshold such that poor hearing (higher BPTA) was more strongly associated with poorer executive function among individuals with less complex social networks, compared to individuals with more complex social networks.

**Table 2:** The moderating effects of social variables on the association between better executive function (PC1) and memory (PC2) and vision and hearing thresholds (higher threshold = worse sensory function).

			PC1				PC2			
			B	SE	t	P	B	SE	t	P
Hearing x	Retirement	Fully retired	Ref.				Ref.			
		Partly retired	**0.048**	**0.022**	**2.170**	**0.030**	−0.010	0.021	−0.470	0.639
		Not retired	−0.004	0.016	−0.260	0.798	**0.030**	**0.015**	**2.020**	**0.043**
	Driving status	Drives at least occasionally	0.046	0.026	1.750	0.081	0.028	0.023	1.260	0.209
	Living situation	Lives alone	−0.008	0.016	−0.510	0.613	0.005	0.015	0.370	0.711
	Social participation	Types	0.002	0.004	0.350	0.725	0.001	0.004	0.340	0.735
		Frequency	0.012	0.011	1.150	0.251	0.008	0.010	0.750	0.455
		Network index	−0.008	0.004	−1.850	0.065	−0.004	0.004	−1.010	0.315
	Life space	Life space index	0.001	0.000	1.860	0.063	0.000	0.000	1.360	0.173
	Social support	Perceived support	0.000	0.000	1.420	0.155	0.000	0.000	0.610	0.544
	Loneliness	Felt lonely in the past week	−0.007	0.015	−0.460	0.645	−0.024	0.014	−1.720	0.085
	Wanted more social participation	Yes	0.005	0.013	0.420	0.676	−0.010	0.013	−0.760	0.448
Vision x	Retirement	Fully retired	Ref.				Ref.			
		Partly retired	0.321	0.214	1.500	0.134	−0.049	0.200	−0.250	0.806
		Not retired	0.021	0.141	0.150	0.884	0.106	0.128	0.830	0.404
	Driving status	Drives at least occasionally	−0.098	0.197	−0.500	0.618	−0.130	0.179	−0.730	0.467
	Living situation	Lives alone	0.157	0.151	1.040	0.298	−0.087	0.142	−0.620	0.537
	Social participation	Types	−0.006	0.043	−0.150	0.883	0.010	0.039	0.260	0.794
		Frequency	0.081	0.104	0.770	0.441	0.119	0.099	1.200	0.231
		Network index	−0.064	0.040	−1.580	0.115	−0.008	0.038	−0.220	0.829
	Life space	Life space index	0.001	0.003	0.230	0.818	**0.007**	**0.003**	**2.410**	**0.016**
	Social support	Perceived support	0.000	0.003	−0.050	0.961	0.002	0.003	0.670	0.501
	Loneliness	Felt lonely in the past week	−0.195	0.145	−1.350	0.178	−0.082	0.137	−0.600	0.548
	Wanted more social participation	Yes	0.018	0.126	0.140	0.885	−0.043	0.122	−0.350	0.726

Shown are the interaction terms of hearing and vision with each of the social variables. The interaction effects are derived from a series of models where one interaction at a time (ten models for interactions with hearing, and ten for interactions with vision) was added to the main effects model shown in Table 2 for PC1 and PC2. Statistically significant effects, in bold, indicate weak but significant (P < 0.05) moderating effects of retirement and SNI on the hearing-PC1 association, LSI on the vision-PC2 association, and retirement on the hearing-PC2 association.

For memory, we observed a *positive*, borderline significant effect of not being retired (relative to being fully retired) on the association between hearing threshold and memory score (Table 2). This, if not a spurious effect, indicates that the general protective effect of better hearing (lower BPTA) on memory was higher in those who were retired relative to those who were not, while the association for those on partial retirement did not differ from those on full retirement.

Finally, we examined social mediation (i.e., the proportion of the sensory-cognitive covariance attributable to an indirect effect of sensory decline on social engagement, which in turn affects cognition). The total variance explained by the social variables was negligible (difference in R^2^ < 0.01 between models including/excluding social variables for both executive function and memory). A comparison of models including and excluding the social variables (Table 3) indicated that 6.8% of the overall effect of hearing and 8.3% of the effect of vision on executive function could be due to the indirect effects of sensory function on social factors (BPTA Δβ = −0.005 dB HL; VA Δβ = −0.065 logMAR). For memory, the indirect effect was 3.7% for hearing (Δβ = −0.002 dB HL). For vision, a 66.9% difference existed between models including and excluding social variables (Δβ = 0.020 logMAR), indicating potentially significant mediating effects of social variables on the association of vision and memory; however, the main effect of vision on memory remained non-significant in the both models (including/excluding the social variables; Table 3). None of these differences between estimates were statistically significant (Z < 0.7, P > 0.5 for all F-tests). These conclusions were qualitatively similar for males and females, and for the younger and older half of the study population (Table 3). However, the Δβ for BPTA was larger for females (15.1%) than for males (0.8%). Both the Δβ-values and the ΔR^2^ were higher for those aged 65 years or above relative to those aged 45–64 years. Notably, twice as much of the variation in executive function was explained by social factors in the older half of the study population (ΔR^2^ = 5.3%) compared to the younger half (ΔR^2^ = 2.6%). Together, these results indicate that the effect of social variables is small overall but may be larger for women and older people, particularly in terms of executive function.

**Table 3:** The mediation effects of hearing (BPTA) and vision (VA) measures on Executive function (PC1) and Memory (PC2) in models with (+social) and without social factors (-social) in the entire dataset (All) and after splitting the data by sex or by age group.

		PC1						PC2					
		+social		−social				+social		−social			
		β	SE	β	SE	Δβ %^a^	Model ΔR^2^%	β	SE	β	SE	Δβ %^b^	Model ΔR^2^%
All	BPTA	−0.077	0.009	−0.082	0.009	−6.8	2.2	−0.046	0.008	−0.048	0.008	−3.7	1.8
	VA	−0.785	0.074	−0.850	0.073	−8.3		−0.029	0.067	−0.049	0.067	−66.9^c^	
Females	BPTA	−0.073	0.011	−0.084	0.013	−15.1	2.4	−0.049	0.012	−0.052	0.012	−5.3	2.0
	VA	−0.791	0.103	−0.848	0.102	−7.2		0.020	0.095	−0.005	0.095	−74.9 ^c^	
Males	BPTA	−0.077	0.013	−0.077	0.011	−0.8	2.0	−0.040	0.011	−0.042	0.011	−4.7	2.8
	VA	−0.776	0.102	−0.854	0.103	−10.0		−0.073	0.093	−0.091	0.092	−23.9^c^	
Age 45–64	BPTA	−0.090	0.013	−0.094	0.013	−4.4	2.6	−0.044	0.012	−0.044	0.012	−0.8	2.4
	VA	−0.853	0.096	−0.896	0.096	−5.0		−0.072	0.091	−0.078	0.091	−8.2^c^	
Age 65–85	BPTA	−0.045	0.011	−0.050	0.011	−9.8	5.3	−0.038	0.010	−0.039	0.010	−3.4	3.0
	VA	−0.636	0.104	−0.733	0.102	−15.2		0.066	0.089	0.024	0.089	−62.8 ^c^	

Note that BPTA and VA were estimated simultaneously in each model. Δβ refers to the percent change in the predicted effect size (β) of BPTA and VA between the pair of models (+social vs. - social) for each group. F-tests indicate that all Δβ-values were statistically nonsignificant. Model ΔR^2^ refers to the approximate change in the variance explained by the + social vs. -social model. The reported adjusted R^2^ values are approximate, and computed without accounting for the survey design (i.e. using unweighted data).

a. F-test: all Z-values ≤ 0.660, P ≫ 0.05

b.F-test: all Z-values ≤ 0.327, P ≫ 0.05

c.Confidence intervals include zero (and |SE| > |β|), i.e. VA has no statistically significant effect on PC2 in either model.

## Discussion

We conducted one of the first evaluations of sensory-social-cognitive associations in an older population without known cognitive impairments (see also^2,9^). We analysed the independent and combined, direct and indirect, effects of multiple aspects of social and sensory variables on cognitive function. The results indicate that age-specific cognitive function is directly associated with both sensory function and social participation. However, we found little support for moderating or mediating effects of social factors on the sensory-cognitive associations in this relatively healthy population. This was true for both sexes and age groups, even though the social factors were slightly more influential in women and older participants, particularly for executive function. Thus, the results do not support the hypothesis that the effects of sensory decline on cognitive decline arise from changes in social participation caused by sensory decline^2,14^. In contrast to the present findings, a population study (men and women aged 50 years or above, sexes analysed together) in the United Kingdom found social mediation between hearing and cognition^9^. The difference in outcomes might be due to the slightly younger population, better average hearing level, and the finer scale measures available from the CLSA compared to the UK study. Overall, it is possible that social isolation secondary to sensory loss may have additive adverse effects on cognition, but that such effects may not manifest until sensory impairments seriously restrict participation or independence. Importantly, our findings show that sensory-cognitive associations begin without mediation by social factors.

Executive function was directly associated with nearly all aspects of social participation, perceived isolation and social opportunity/independence, whereas memory scores were associated with perceived social support and weakly with aspects of social opportunity (retirement, life space, driving). Most of the effects were very small, but their compounded effects suggest an important connection between social participation and cognition in community-dwelling older adults, consistent with previous findings^28–30^.

Interestingly, the highest cognitive scores were seen for partially retired (partially working) participants compared to those who were not retired or fully retired. Retirement status was also one of the few statistically significant moderators: better executive function was found in those with poorer hearing (higher BPTA) if they were partially retired and better memory was found in those with poorer hearing (higher BPTA) if they were not retired relative to those who were fully or partially retired. These findings might suggest that participants who were partially retired experienced a more complex social environment and had more adept coping mechanisms or more accommodating environments, which phenomena merit further study. Although the proportion of partially retired participants (11%) was small relative to fully retired (45%) and non-retired participants (44%), the special status of partial retirement is unlikely to be a statistical artefact, and aligns with findings suggesting that cognitive and physical health declines are alleviated by part-time work and social activity after retirement^31^.

In addition to social factors, physical inactivity and depression have been identified as important predictors of dementia, possibly in interaction with sensory and social aspects^10,32^. In the CLSA, loneliness and desire to engage in more activities were associated with lower life satisfaction and a higher depression risk^33^. A limitation of our study and others is that the good cognitive and sensory health of the population reduces variability in the estimates, and the cross-sectional design limits examination of causation. Further investigation such as path analysis using longitudinal data is required to investigate the causal pathways between sensory, cognitive, and social factors, and to test other hypotheses and mediators for sensory-social-cognitive associations^14^. As sensory and cognitive impairments increase in prevalence and severity with age, an improved understanding of these associations will help support older adults’ independence and quality of life.

Appendix: Supplementary information contains additional information on data processing, descriptive data and full model outputs.

## Supplementary information

Appendix

## Data Availability

The data that support the findings of this study are available from the Canadian Longitudinal Study of Aging (CLSA) but restrictions apply to the availability of these data, which were used under license and data-sharing agreement for the current study, and so are not publicly available.
