# Supplementary material for: Sensory-cognitive associations are only weakly mediated or moderated by social factors in the Canadian Longitudinal Study on Aging
Source: Sci Rep. 2019 Dec 23;9:19660. doi: 10.1038/s41598-019-55696-5 (PMC6928150; doi:10.1038/s41598-019-55696-5)
Supplement: Supplementary file 1 — Appendix [file 41598_2019_55696_MOESM1_ESM.docx]

APPENDIX

Sensory-cognitive associations are only weakly mediated or moderated by social factors in the Canadian Longitudinal Study on Aging

Anni Hämäläinen^1^, Natalie Phillips^2^, Walter Wittich^1^, M. Kathleen Pichora-Fuller^3^, Paul Mick^4^*

1. School of Optometry, Université de Montréal
2. Department of Psychology, Concordia University
3. Department of Psychology, University of Toronto
4. Department of Surgery, University of Saskatchewan

Contents

[Supplemental information on methodology 2](#_Toc18166699)

[Multiple imputation approach 2](#_Toc18166700)

[Two-step imputation approach overview 2](#_Toc18166701)

[Mediation analysis 5](#_Toc18166702)

[References 5](#_Toc18166703)

[Supplemental tables 7](#_Toc18166704)

[Table S1: Descriptive data on all variables used in the analyses. 7](#_Toc18166705)

[Table S2: Full main-effects models including social variables for executive function (PC1) and memory (PC2). 9](#_Toc18166706)

[Table S3: Main effects models excluding social variables for executive function (PC1) and memory (PC2), used for assessing the combined mediation effect of social variables. 11](#_Toc18166707)

[Table S4: Mean adjusted R^2^-values for models with and without social factors, calculated with *mibeta* (not accounting for survey design) for the complete imputed data set and split by sex and age group. 12](#_Toc18166708)

# Supplemental information on methodology

## Multiple imputation approach

The analyses required deriving a principal component score to assess cognitive performance, and using the principal component scores to examine risk factors for poor cognitive performance using multiple regression models. Thus, we used a two-step multiple imputation approach to maximize the use of available data collected from the participants on a large number of variables. In Step 1, we derived principal component scores via **mvn** multiple imputation using all available cognitive test results. In Step 2, we imputed missing data in these PC scores and all other variables of interest using a **chained-equations** multiple imputation step.

### Two-step imputation approach overview

Multiple imputation was done in two stages: 1) multivariate normal regression imputation to generate a covariance matrix for principal component analysis of the cognitive variables; and 2) chained-equations multiple imputation (M=30 imputed datasets) to derive estimates for the missing values in the composite cognitive scores and all independent variables, and used these values in the multiple imputation framework to estimate all multiple regression models.

This two-step imputation approach was chosen because 1) the cumulative proportion of missing data for the cognitive tests was relatively high (approximately 17%) although any one participant rarely lacked data for more than one test; and 2) it is recommended that the “final” form of the variables of interest be included in the multiple imputation process (here: PC-scores rather than the raw data; see e.g. ^1^; <https://stats.idre.ucla.edu/stata/seminars/mi_in_stata_pt1_new>).

#### Step 1: Principal component analysis of cognitive variables with multiple imputation

We derived an imputed correlation matrix of the seven cognitive test results using a maximum likelihood estimation with an expectation-maximization (EM) algorithm in Stata’s *mi* framework (<https://stats.idre.ucla.edu/stata/faq/how-can-i-do-factor-analysis-with-missing-data-in-stata/>; ^2–5^. As recommended in the above tutorial, we assumed an arbitrary sample size for this estimation as the N of non-missing observations for the variable with the largest proportion of missing data - in this case, the mental alternation test (N=27,615). This imputed correlation matrix was then used to generate the component "weights" for each variable included in the PCA and used to compute the principal component scores (see PC scores below).

##### Cognitive test results

The cognitive tests used to compute the cognitive scores were:

- **Memory:**
  - **Rey Auditory Verbal Learning Test.** Participants listen to a recorded list of 15 words once, and are asked to repeat the words back immediately and again 5 min. Two variables were retained for analyses: **Delayed recall** (REY2; # words correctly recalled 5 min after hearing the recording) and **Retention** (REYr; ratio of delayed to immediate recall).
- **Executive function.**
  - **Controlled Oral Word Association Test.** Participant is asked to generate as many words as possible that begin with a given letter in 60s (3 trials, letters F, A, S). Test score = total number of words (FAS).
  - **Animal fluency test.** Participant is asked to generate as many animals as possible in 60 s. Test score = total number of different animals named (AFT).
  - **Mental alternation test.** Participant is asked to generate the numbers 1–26 and the letters of the alphabet, alternating between each (1-A, 2-B, 3-C, ...). Test score = number of correct alternations (MAT).
  - **Stroop test Victoria version.** Participant is asked to quickly name the color of a visual cue (dots or words on cards). Two variables were retained for analyses: **Interference score** (STP; total time taken to name the ink colors, rather than the color word printed on a card) and **Stroop ratio** (STPr; ratio of Interference score to Control task score, naming the color of printed dots.

Details on the choice and administration of the cognitive tests are provided in ^6,7^, and the detailed method for deriving the cognitive scores through principal component analyses are provided in ^8^, with a brief description provided below.

##### PC scores

After extracting the principal components, we calculated the PC scores for those individuals who had complete cognitive data available and no diagnosed dementia (i.e., for a sample size of N=25 011 there were N=5018 participants missing). The scores used for the PC estimation are thus based on the imputed correlation matrix rather than the raw data.

Rotated loadings (orthogonal varimax rotation with Kaiser correction) estimated using the EM algorithm indicated that the first axis captures mostly variance in the executive function test results (high loadings of AFT, FAS, MAT, STP and STPr, Eigenvalue = 2.4, explained 34.5% of the variation in the data) and variables associated with memory loaded strongly on the second axis (REY2, REYr, Eigenvalue = 1.8, 25.2% of total variance explained). The combined variance explained by these two components was 59.7%. Thus, we extracted the scores for PC1, taken to represent Executive function, and PC2, taken to represent Memory. The weights for PC1 and PC2 derived from the EM-algorithm were used to calculate PC1 and PC2 scores for those participants who had existing data for all 7 test results and those participants with missing data for any of the 7 variables had missing data for both PC1 and PC2. Finally, we imputed the missing data in these principal components in the chained-equations multiple imputation (see Step 2).

Rotated loadings estimated using the EM algorithm indicated that the first axis captures mostly variance in the executive function test results (5 variables derived from 4 tests) and variables associated with memory (2 variables derived from one two-trial test) loaded strongly on the second axis. Thus, we extracted the scores for PC1, taken to represent Executive function, and PC2, taken to represent Memory. The weights for PC1 and PC2 derived from the EM-algorithm were used to calculate PC1 and PC2 scores for those participants who had existing data for all 7 test results and those participants with missing data for any of the 7 variables had missing data for both PC1 and PC2. We then included these principal components in the global, multivariate multiple imputation method to impute the missing cognitive scores (PC1 and PC2).

####

#### Step 2: Chained-equations multiple imputation method

A moderate amount of data was missing for some variables (Table S1). Combined with the large number of variables included in our models, the additive number of observations excluded from complete case analyses came to >50% for some models (see “Complete case analyses” below). We also have reason to assume that the data are “missing at random” (“MAR”) (i.e., the probability that data are missing depends, at least in part, on other, observed data). For example, exploratory analyses suggested that those with missing data on income, hearing and cognition may be more likely to be older and less healthy (see also Mick et al. unpublished), leading to misrepresentation of the sample when using complete-case analyses (StataCorp LLC 2017 and literature therein). We therefore chose to conduct multiple imputations to take full advantage of all available data in our analyses.

We used a chained-equations multiple imputation approach (“MICE”) to make use of non-missing data to estimate the missing values for all variables with iterative multivariate models. For general information on the method, see ^11–13^ and for detailed advice on the implementation of the method in Stata, see <https://stats.idre.ucla.edu/stata/seminars/mi_in_stata_pt1_new/>. Briefly, MICE is an iterative multivariate approach in which the missing data for each variable are imputed in turn, starting with the variable with the least missing data. The imputations are done through regressions of each variable against all other variables in the imputation run, such that different data distributions can be modeled using the appropriate error structure. Thus, the missing values for each of the variables included in the imputation run are predicted using all other variables in the same run. For example, missing values for Income were derived from a model that used Income as a dependent variable, and all other variables as independent variables. This iterative process is used to generate a number of imputed datasets, which are then used for follow-up analyses (here: deriving parameter estimates from multiple regression models). The error associated with the estimation of the missing values is captured in the slight differences in estimates among imputed datasets. The slight differences among the datasets are used during model estimation to adjust the standard errors for each of the variables that had any missing data. This is done by adjusting (increasing) the standard errors around the estimates, and these upscaled standard errors are then used in the calculation of the statistical significance of the associations between variables. All estimates reported in the main body of the paper were based on these imputed and error-adjusted estimates.

We used MICE to derive estimates for the missing values in all variables of interest. In addition to the variables used as independent and dependent variables in our multiple regression models, we included auxiliary variables (as long as the additional variables did not prevent convergence of the imputations) that were not included in the models of self-reported sensory difficulties, but could improve the imputed estimates for variables of interest. To this end, we chose auxiliary variables that were correlated with the variables of interest, and/or could provide information about the true values of the missing data, or about the likelihood of missingness for a variable.

We generated M=30 imputed datasets (recent recommendation for minimum M=20 to reduce sampling error due to imputations; ^10^. We used the default burn-in period of 10 iterations per dataset (5-20 recommended, e.g. ^14^ (i.e., 300 iterations were used to obtain the 30 datasets). After completing the imputations, the resulting 30 complete datasets (with complete data for all variables for all 30,029 rows (i.e., individuals) in each of the datasets) were used to estimate the models for the variables of interest using Rubin’s rules ^15^.

To evaluate the performance of the multiple-imputed data (i.e., adjusting for uncertainty from missing data), we performed sensitivity analyses by comparing the results of our imputed models to the same models using complete cases. Additionally, we performed various diagnostics as recommended by e.g. ^10,16,17^; see also <https://stats.idre.ucla.edu/stata/seminars/mi_in_stata_pt1_new/>. We used diagnostic plots to check the variables with the most missing data for consistency relative to observed data and examined the trace plots and autocorrelation plots to assess convergence. Furthermore, we assessed the relative efficiencies, within-and between-data set variances and inflation of standard errors to assess the adequacy of the imputations and their effects on the model outcomes. We also examined the Fraction of Missing Information (FMI; proportion of the total sampling variance that is due to missing data); the Relative Increase in Variance (RVI; proportion of *increase* in total sampling variance due to missing information); the Relative Efficiency (RE; estimated efficiency of the imputation, relative to performing an infinite number of imputations), as well as the between-imputation and the within-imputation variance estimates for all variables used in the models. The diagnostics identified no issues with the imputations. The increase in standard errors due to missing data ranged from 6-21% for PC1 and 7-23% for PC2, but with 30 imputed datasets, the relative efficiencies were ≥0.99 throughout, suggesting that the results based on these datasets are reliable.

## Mediation analysis

In the paper, we explored the possibility that the association between vision and/or hearing could be mediated by the overall social participation status of the person by comparing models that included all available social variables with models that excluded all social variables. This constitutes a so-called causal steps approach as described by Fairchild and McDaniel ^18^. Using those nested models, we quantified the strength of the association between vision/hearing and memory/executive function and determined how much the strength of that association changed, i.e. whether the strength of the association was reduced dues to social variables. A shortcoming of this mediation testing approach is the lack of a formal significance test ^18^. We chose to mitigate this problem by estimating the statistical significance of the difference in effect sizes between the nested models (with and without social variables) with Z-tests. It has been pointed out that strong causal inference should not be made from cross-sectional data ^18,19^; thus the outcomes of our mediation tests should be treated with caution.

## References

1. von Hippel, P. T. How To Impute Squares, Interactions, and Other Transformed Variables. *Sociol. Methodol.* **39,** 265–291 (2009).

2. Truxillo, C. Maximum likelihood parameter estimation with incomplete data. *Proc. Thirtieth Annu. SAS® Users Group.* 1–19 (2005).

3. Graham, J. W. Missing Data Analysis: Making It Work in the Real World. *Annu. Rev. Psychol.* **60,** 549–576 (2009).

4. Weaver, B. & Maxwell, H. Exploratory factor analysis and reliability analysis with missing data: A simple method for SPSS users. *Quant. Methods Psychol.* **10,** 143–152 (2014).

5. Schafer, J. L. *Analysis of Incomplete Multivariate Data*. (Chapman and Hall/CRC, 1997).

6. Tuokko, H., Griffith, L. E., Simard, M. & Taler, V. Cognitive measures in the Canadian Longitudinal Study on Aging. *Clin. Neuropsychol.* **31,** 233–250 (2017).

7. Raina, P., Wolfson, C. & Kirkland, S. *The Canadian Longitudinal Study on Aging (CLSA) Report on Health and Aging in Canada: Findings from Baseline Data Collection 2010-2015. Prepared for the Canadian Institutes for Health Research and the Government of Canada, August 2018.* (2018).

8. Phillips, N., Hämäläinen, A., Wittich, W., Pichora-Fuller, M. K. & Mick, P. Associations between cognitive and sensory function in older adults: a snapshot of the first wave of the Canadian Longitudinal Study of Aging (CLSA). I*n preparation.*

9. Mick, P. *et al.* The prevalence of hearing and vision loss in older Canadians: An analysis of Data from the Canadian Longitudinal Study on Aging. *In review.*

10. StataCorp LLC. Stata multiple-imputation reference manual. (2017).

11. Royston, P. & White, I. Multiple Imputation by Chained Equations (MICE): Implementation in Stata. *J. Stat. Softw.* **45,** 1–20 (2011).

12. White, I. R., Royston, P. & Wood, A. M. Multiple imputation using chained equations: Issues and guidance for practice. *Stat. Med.* **30,** 377–399 (2011).

13. Azur, M. J., Stuart, E. A., Frangakis, C. & Leaf, P. J. Multiple Imputation by Chained Equations: What is it and how does it work? *Int J Methods Psychiatr Res* **20,** 40–49 (2011).

14. Buuren, S. Van. Multiple imputation of discrete and continuous data by fully conditional specificatio. *Stat. Methods Med. Res.* **16,** 219–242 (2007).

15. Rubin, D. B. *Multiple Imputation for Nonresponse in Surveys.* (J. Wiley & Sons, 1987).

16. Nguyen, C. D., Carlin, J. B. & Lee, K. J. Model checking in multiple imputation : an overview and case study. *Emerg. Themes Epidemiol.* 1–12 (2017). doi:10.1186/s12982-017-0062-6

17. Abayomi, K., Gelman, A. & Levy, M. Diagnostics for Multivariate Imputations. *J. R. Stat. Soc. Ser. C (Applied Stat.* **57,** 273–291 (2008).

18. Fairchild, A. J. & Mcdaniel, H. L. Statistical Commentary Best (but oft-forgotten) practices: mediation analysis 1,2. *Am J Clin Nutr* **105,** 1259–71 (2017).

19. Vanderweele, T. J. Invited commentary: Structural equation models and epidemiologic analysis. *Am. J. Epidemiol.* **176,** 608–612 (2012).

# Supplemental tables

## Table S1: Descriptive data on all variables used in the analyses.

|  |  | **Unimputed N** | **Mean** | **SD** | **Min** | **Max** | **%** |
| --- | --- | --- | --- | --- | --- | --- | --- |
| Hearing threshold | Better ear PTA (dB HL) | 27,065 | 18.5 | 13.6 | 0.0 | 102.5 |  |
| Visual acuity | Better eye pinhole-corrected logMAR | 29,319 | 0.1 | 0.1 | -0.3 | 1.1 |  |
| Age | Years | 30,029 | 62.9 | 10.2 | 45 | 86 |  |
| Sex | %female | 30,029 | 0.5 | 0.5 | 0 | 1 | 50.9 |
| Education (higher = more education) | | 29,955 | 1.4 | 0.9 | 1 | 4 |  |
|  | Post-graduate degree | 23,267 |  |  |  |  | 77.7 |
|  | Some post-secondary education | 2,226 |  |  |  |  | 7.4 |
|  | Secondary school graduate | 2,828 |  |  |  |  | 9.4 |
|  | < secondary school graduation | 1,634 |  |  |  |  | 5.5 |
| Household income (higher= larger annual household income) | | 28,100 | 2.8 | 1.1 | 1 | 5 |  |
|  | >= $150,000 | 4,792 |  |  |  |  | 17.1 |
|  | $100,000-149,999 | 5,514 |  |  |  |  | 19.6 |
|  | $50,000-99,999 | 9,890 |  |  |  |  | 35.2 |
|  | $20,000-49,999 | 6,341 |  |  |  |  | 22.6 |
|  | <$20,000 | 1,563 |  |  |  |  | 5.6 |
| Cultural background (non-white, white) | %white | 30,013 | 0.9 | 0.2 | 0 | 1 | 94.2 |
| Test language (English, French, Bilingual) | | 29,590 | 0.3 | 0.6 | 0 | 2 |  |
|  | English | 23,188 |  |  |  |  | 78.4 |
|  | French | 4,812 |  |  |  |  | 16.3 |
|  | Bilingual (switched language) | 1,590 |  |  |  |  | 5.4 |
| Hypertension (0=no, 1=yes) | %yes | 29,834 | 0.4 | 0.5 | 0 | 1 | 37.1 |
| Nutritional status (1=High risk) | %High nutritional risk | 28,397 | 0.4 | 0.5 | 0 | 1 | 64.5 |
| Diabetes (0=no, 1=yes) | %yes | 29,903 | 0.2 | 0.4 | 0 | 1 | 17.7 |
| Smoking status (higher = current regular smoker) | | 29,934 | 1.7 | 0.8 | 1 | 4 |  |
|  | Never | 14,227 |  |  |  |  | 47.5 |
|  | Former smoker | 13,146 |  |  |  |  | 43.9 |
|  | Current occasional smoker | 483 |  |  |  |  | 1.6 |
|  | Current daily smoker | 2,078 |  |  |  |  | 6.9 |
| Body mass index | BMI | 29,877 | 28.1 | 5.4 | 12.9 | 69.7 |  |
| Head injuries | %at least one head injury | 30,029 | 0.2 | 0.4 | 0 | 1 | 24.2 |
| Retirement status | Completely retired | 13,320 |  |  |  |  | 44.5 |
|  | Partly retired | 3,308 |  |  |  |  | 11.1 |
|  | Not retired | 13,292 |  |  |  |  | 44.4 |
| Driving status | %drives at least occasionally | 27,504 | 0.9 | 0.3 | 0 | 1 | 92.2 |
| Living arrangement | %Lives alone | 29,986 | 0.2 | 0.4 | 0 | 1 |  |
| Social participation | Participation types count | 29,967 | 2.2 | 1.5 | 0 | 8 |  |
|  | Participation frequency ordinal | 29,958 | 3.0 | 0.6 | 0 | 4 |  |
|  | Network index (increasing) | 29,925 | 4.3 | 1.6 | 0 | 10 |  |
| Social opportunity | Life space index | 29,976 | 85.3 | 18.2 | 0 | 120 |  |
| Social support | Perceived support | 29,940 | 101.9 | 22.1 | 0 | 125 |  |
| Loneliness | %Sometimes-all the time (>1d) | 29,910 | 0.3 | 0.4 | 0 | 1 | 26.0 |
| Wanted more social participation | %yes | 29,936 | 0.5 | 0.5 | 0 | 1 | 47.7 |
| Executive function |  | 25,011 | 1.3 | 1.5 | -9.6 | 6.8 |  |
| Memory |  | 25,011 | 4.4 | 1.3 | 0.5 | 9.1 |  |

## Table S2: Full main-effects models including social variables for executive function (PC1) and memory (PC2).

|  |  | PC1 |  |  |  | PC2 |  |  |  |
| --- | --- | --- | --- | --- | --- | --- | --- | --- | --- |
|  |  | B | SE | t | P | B | SE | t | P |
| Hearing threshold | Better ear PTA (10 dB HL) | -0.077 | 0.009 | -8.960 | <0.001 | -0.046 | 0.008 | -5.980 | <0.001 |
| Visual acuity | Better eye pinhole-corrected logMAR | -0.785 | 0.074 | -10.670 | <0.001 | -0.029 | 0.067 | -0.440 | 0.660 |
| Age | Years | -0.039 | 0.002 | -25.280 | <0.001 | -0.029 | 0.001 | -20.300 | <0.001 |
| Education | Post-graduate degree | Ref. |  |  |  | Ref. |  |  |  |
|  | Some post-secondary education | -0.124 | 0.033 | -3.790 | <0.001 | -0.091 | 0.033 | -2.760 | 0.006 |
|  | Secondary school graduate | -0.362 | 0.031 | -11.610 | <0.001 | -0.233 | 0.032 | -7.330 | <0.001 |
|  | < secondary school graduation | -0.838 | 0.046 | -18.410 | <0.001 | -0.390 | 0.041 | -9.440 | <0.001 |
| Household income | >=$150,000 | Ref. |  |  |  | Ref. |  |  |  |
|  | $100,000-149,999 | -0.124 | 0.028 | -4.490 | <0.001 | 0.017 | 0.028 | 0.600 | 0.551 |
|  | $50,000-99,999 | -0.248 | 0.028 | -8.810 | <0.001 | -0.041 | 0.027 | -1.550 | 0.121 |
|  | $20,000-49,999 | -0.508 | 0.035 | -14.590 | <0.001 | -0.133 | 0.034 | -3.930 | <0.001 |
|  | <$20,000 | -0.625 | 0.057 | -11.070 | <0.001 | -0.156 | 0.052 | -2.980 | 0.003 |
| Sex | Male | -0.056 | 0.019 | -2.990 | 0.003 | -0.445 | 0.018 | -24.800 | <0.001 |
| Cultural background | White | 0.596 | 0.040 | 14.850 | <0.001 | 0.182 | 0.039 | 4.650 | <0.001 |
| Test language | English | Ref. |  |  |  | Ref. |  |  |  |
|  | French | -0.408 | 0.026 | -15.860 | <0.001 | 0.022 | 0.024 | 0.940 | 0.346 |
|  | Bilingual (switched language) | -0.129 | 0.043 | -2.990 | 0.003 | 0.038 | 0.040 | 0.950 | 0.345 |
| Hypertension | Yes | -0.092 | 0.021 | -4.460 | <0.001 | -0.034 | 0.020 | -1.710 | 0.087 |
| Nutritional status | High nutritional risk | -0.080 | 0.020 | -3.930 | <0.001 | -0.052 | 0.020 | -2.560 | 0.011 |
| Diabetes | Yes | -0.058 | 0.025 | -2.290 | 0.022 | -0.031 | 0.023 | -1.310 | 0.189 |
| Smoking status | Never | Ref. |  |  |  | Ref. |  |  |  |
|  | Former smoker | 0.004 | 0.019 | 0.240 | 0.813 | -0.034 | 0.018 | -1.930 | 0.054 |
|  | Current occasional smoker | -0.080 | 0.070 | -1.140 | 0.255 | -0.004 | 0.070 | -0.050 | 0.960 |
|  | Current daily smoker | -0.084 | 0.039 | -2.160 | 0.031 | -0.080 | 0.036 | -2.200 | 0.028 |
| Body mass | BMI | -0.010 | 0.002 | -5.580 | <0.001 | -0.001 | 0.002 | -0.390 | 0.695 |
| Head injuries | At least one head injury | 0.045 | 0.020 | 2.250 | 0.025 | 0.060 | 0.018 | 3.310 | 0.001 |
| Retirement status | fully retired | Ref. |  |  |  | Ref. |  |  |  |
|  | partly retired | 0.158 | 0.030 | 5.260 | <0.001 | 0.072 | 0.030 | 2.420 | 0.016 |
|  | not retired | 0.050 | 0.028 | 1.780 | 0.075 | -0.036 | 0.026 | -1.350 | 0.177 |
| Driving status | Drives at least occasionally | 0.163 | 0.043 | 3.780 | <0.001 | 0.092 | 0.039 | 2.360 | 0.019 |
| Living arrangement | Lives alone | 0.130 | 0.027 | 4.820 | <0.001 | 0.020 | 0.026 | 0.790 | 0.431 |
| Social participation | Participation types | 0.037 | 0.008 | 4.480 | <0.001 | 0.008 | 0.008 | 0.910 | 0.365 |
|  | Participation frequency | -0.023 | 0.018 | -1.290 | 0.196 | -0.009 | 0.018 | -0.520 | 0.603 |
|  | Network index | -0.009 | 0.007 | -1.240 | 0.215 | 0.005 | 0.008 | 0.650 | 0.517 |
| Social opportunity | Life space index | 0.002 | 0.001 | 4.010 | <0.001 | -0.002 | 0.001 | -2.970 | 0.003 |
| Social support | Perceived support | 0.002 | 0.001 | 3.190 | 0.001 | 0.002 | 0.000 | 3.490 | <0.001 |
| Loneliness | Sometimes-all the time (>1d) | -0.071 | 0.024 | -2.980 | 0.003 | -0.042 | 0.023 | -1.830 | 0.068 |
| Wanted more social participation | Yes | 0.045 | 0.018 | 2.530 | 0.011 | 0.008 | 0.018 | 0.460 | 0.644 |
| Intercept |  | 3.596 | 0.147 | 24.400 | <0.001 | 6.396 | 0.143 | 44.760 | <0.001 |

## Table S3: Main effects models excluding social variables for executive function (PC1) and memory (PC2), used for assessing the combined mediation effect of social variables.

|  |  | PC1 |  |  |  | PC2 |  |  |  |
| --- | --- | --- | --- | --- | --- | --- | --- | --- | --- |
|  |  | B | SE | t | P | B | SE | t | P |
| Hearing threshold | Better ear PTA (10 dB HL) | -0.082^1^ | 0.009 | -9.530 | <0.001 | -0.048^2^ | 0.008 | -6.200 | <0.001 |
| Visual acuity | Better eye pinhole-corrected logMAR | -0.850^3^ | 0.073 | -11.580 | <0.001 | -0.049^4^ | 0.067 | -0.730 | 0.464 |
| Age | Years | -0.040 | 0.001 | -29.930 | <0.001 | -0.027 | 0.001 | -23.100 | <0.001 |
| Education | Post-graduate degree | Ref. |  |  |  | Ref. |  |  |  |
|  | Some post-secondary education | -0.139 | 0.033 | -4.230 | <0.001 | -0.094 | 0.033 | -2.860 | 0.004 |
|  | Secondary school graduate | -0.373 | 0.031 | -11.960 | <0.001 | -0.233 | 0.032 | -7.310 | <0.001 |
|  | < secondary school graduation | -0.872 | 0.045 | -19.170 | <0.001 | -0.393 | 0.041 | -9.560 | <0.001 |
| Household income | >=$150,000 | Ref. |  |  |  | Ref. |  |  |  |
|  | $100,000-149,999 | -0.129 | 0.027 | -4.710 | <0.001 | 0.017 | 0.028 | 0.610 | 0.539 |
|  | $50,000-99,999 | -0.261 | 0.028 | -9.470 | <0.001 | -0.044 | 0.026 | -1.660 | 0.097 |
|  | $20,000-49,999 | -0.534 | 0.033 | -16.220 | <0.001 | -0.148 | 0.032 | -4.590 | <0.001 |
|  | <$20,000 | -0.692 | 0.051 | -13.480 | <0.001 | -0.201 | 0.048 | -4.140 | <0.001 |
| Sex | Male | -0.050 | 0.018 | -2.740 | 0.006 | -0.451 | 0.017 | -25.850 | <0.001 |
| Cultural background | White | 0.629 | 0.040 | 15.710 | <0.001 | 0.196 | 0.039 | 5.010 | <0.001 |
| Test language | English | Ref. |  |  |  | Ref. |  |  |  |
|  | French | -0.412 | 0.026 | -16.110 | <0.001 | 0.018 | 0.023 | 0.790 | 0.431 |
|  | Bilingual (switched language) | -0.126 | 0.044 | -2.890 | 0.004 | 0.036 | 0.040 | 0.900 | 0.368 |
| Hypertension | Yes | -0.098 | 0.021 | -4.720 | <0.001 | -0.034 | 0.020 | -1.730 | 0.084 |
| Nutritional status | High nutritional risk | -0.101 | 0.020 | -5.080 | <0.001 | -0.068 | 0.020 | -3.400 | 0.001 |
| Diabetes | Yes | -0.066 | 0.025 | -2.610 | 0.009 | -0.029 | 0.023 | -1.250 | 0.211 |
| Smoking status | Never | Ref. |  |  |  | Ref. |  |  |  |
|  | Former smoker | 0.005 | 0.019 | 0.290 | 0.775 | -0.034 | 0.018 | -1.890 | 0.058 |
|  | Current occasional smoker | -0.092 | 0.071 | -1.290 | 0.196 | -0.008 | 0.071 | -0.110 | 0.911 |
|  | Current daily smoker | -0.119 | 0.039 | -3.070 | 0.002 | -0.092 | 0.036 | -2.550 | 0.011 |
| Body mass | BMI | -0.010 | 0.002 | -5.570 | <0.001 | 0.000 | 0.002 | -0.170 | 0.868 |
| Head injuries | At least one head injury | 0.042 | 0.020 | 2.090 | 0.037 | 0.059 | 0.018 | 3.210 | 0.001 |
| Intercept |  | 4.186 | 0.094 | 44.520 | <0.001 | 6.382 | 0.090 | 70.830 | <0.001 |

1. To derive mediation effect, compare effect size to B of visual acuity on PC1 in main text Table 1
2. To derive mediation effect, compare effect size to B of visual acuity on PC2 in main text Table 1
3. To derive mediation effect, compare effect size to B of hearing threshold on PC1 in main text Table 1
4. To derive mediation effect, compare effect size to B of hearing threshold on PC2 in main text Table 1

## Table S4: Mean adjusted R^2^-values for models with and without social factors, calculated with *mibeta* (not accounting for survey design) for the complete imputed data set and split by sex and age group.

|  | Response | R^2^, social | R^2^, no social | ΔR^2^ | ΔR^2^ % |
| --- | --- | --- | --- | --- | --- |
| All | PC1 | 0.29 | 0.28 | 0.006 | 2.23 |
|  | PC2 | 0.14 | 0.13 | 0.002 | 1.77 |
| Females | PC1 | 0.29 | 0.29 | 0.007 | 2.39 |
|  | PC2 | 0.10 | 0.10 | 0.002 | 1.97 |
| Males | PC1 | 0.28 | 0.27 | 0.006 | 2.03 |
|  | PC2 | 0.12 | 0.11 | 0.003 | 2.83 |
| Age 45-64 | PC1 | 0.16 | 0.16 | 0.004 | 2.61 |
|  | PC2 | 0.07 | 0.06 | 0.002 | 2.39 |
| Age 65-85 | PC1 | 0.20 | 0.18 | 0.010 | 5.26 |
|  | PC2 | 0.11 | 0.10 | 0.003 | 3.03 |
